# AD genes and microglia phenotypes: does microglia phenotypic heterogeneity matter?

**DOI:** 10.1002/alz.093420

**Published:** 2025-01-03

**Authors:** Marta Olah

**Affiliations:** ^1^ The Taub Institute for Research on Alzheimer’s Disease and the Aging Brain, New York, NY, USA; Columbia University Irving Medical Center, New York, NY USA

## Abstract

The view of microglial role in the pathogenesis of Alzheimer’s disease (AD) has been shaped by two major breakthroughs in the past decade: the discovery that many of the AD susceptibility genes are preferentially expressed in microglia, and the finding that microglia exist in a variety of previously not described functional phenotypes in the aged human central nervous system. As much as these discoveries brought paradigm shifts to the field, they also revealed major gaps in our understanding of the functional consequences of both the expression of AD susceptibility genes and microglia phenotypic heterogeneity. While there is progress being made in understanding the role of individual AD susceptibility genes in microglia function and what role the different microglia phenotypes might play in disease pathogenesis in AD, the connection between these two determining factors has not been established yet. In order to explore the relationship between the expression of AD susceptibility genes and the different microglia phenotypes that have been described in the aged and AD human brain, we explored the protein expression levels of AD relevant markers in a microglia subset specific way. Our findings highlight the importance of considering the phenotypic heterogeneity of microglia in the course of functional genomic studies and for any future disease modifying therapies targeting AD susceptibility genes in microglia.

